# Preparation of Paralympic Athletes; Environmental Concerns and Heat Acclimation

**DOI:** 10.3389/fphys.2015.00415

**Published:** 2016-01-14

**Authors:** Mike J. Price

**Affiliations:** Faculty of Health and Life Sciences, School of Life Sciences, Applied Biology and Exercise Science Research Centre, Coventry UniversityCoventry, UK

**Keywords:** spinal cord injury, paraplegia, tetraplegia, exercise, performance, heat, humidity

High ambient temperature and relative humidity (rh) are of great importance when considering athletic performance. Such factors are of particular interest when considering that the majority of Paralympic Games have been hosted at locations with potentially challenging environmental conditions (e.g., Atlanta, Sydney, Athens, Beijing) and a range of Paralympic athletes exhibit conditions manifesting thermal dysfunction. Prior to the Atlanta Games in 1996, Nielsen (1996) considered the “fight against physics” with respect to high ambient temperature (30–38°C) and relative humidity (rh; 40–80%) on endurance performance for able-bodied athletes. It was noted that with such potentially severe conditions outdoor endurance based performances could be severely reduced, especially in spells of high humidity. Strategies to prevent heat illness were recommended including events being scheduled at times of lower thermal stress or re-scheduled if temperatures were above 35°C. However, how these conditions may affect Paralympic athletes has not yet been reported. This article will consider what is known regarding enhancing (or maintaining) performance in the heat in athletes with motor disabilities in preparation for Rio 2016.

## Likely conditions for Rio, 2016

Average daily environmental temperatures expected for Rio during August are ~28°C. However, at the time of writing (August, 2015), peak daily environmental temperatures at the four Paralympic venues (Deodoro, Maracana, Copacabana, and Barra) were between 32 and 36°C (35–50% rh) at 12:00–15:00 h. Furthermore, peak humidity rose to 70–75% during early to late evening (18:00–21:00 h) with some early morning humidity values reaching 100% (Worldwideweather, August 2015)^1^. As optimal environmental temperatures for endurance performance in those with intact thermoregulatory systems are between 6–10°C (Galloway and Maughan, 1997) and uncompensable heat stress occurs at environmental temperatures of ~35°C and >60% rh (Nielsen, 1996) these conditions are challenging at best. For Paralympic athletes with thermal dysfunction performance, as well as daily activities and health, may well be negatively affected.

## Paralympic populations at risk

Paralympic classifications for competition include spinal cord impairment, visual impairment, cerebral palsy, amputees, and Les Autres. Athletes with motor disabilities are reflected across a number of classifications. Contributing causes of motor disabilities include traumatic (spinal cord injury or loss of limbs) and disease or congenital conditions (cerebral palsy, muscular dystrophy, multiple sclerosis, or spina bifida). The greatest amount of literature pertaining to thermoregulatory responses during exercise for any group of Paralympic related conditions is for athletes with spinal cord injury (SCI) who demonstrate both motor and neurological deficits. This body of literature most likely reflects the clear thermal dysfunction of this population in proportion to the level of spinal cord injury (For review see Price, 2006). Literature concerning thermoregulatory responses of other Paralympic conditions is unfortunately lacking.

Spinal cord injury results in a loss of motor function and neurological innervation below the level of injury (Price, 2006). In general, athletes with paraplegia (i.e., thoracic and lumbar spinal injuries) demonstrate reduced recruitable muscle mass and a sympathetic nervous system in proportion to the level of lesion. Those athletes with tetraplegia (i.e., cervical spinal injuries) demonstrate the smallest amount of recruitable muscle mass, characterized by upper limb dysfunction due to the injury occurring at a level within the brachial plexus which serves the upper limb. Athletes with tetraplegia also generally demonstrate no sympathetic nervous system innervation due to the level of injury also being above the thoracolumbar sympathetic outflow. As the key thermoregulatory effectors for heat dissipation, namely sweating, and changes in cutaneous blood flow, are sympathetically driven athletes with tetraplegia demonstrate an absent or much reduced sweating capacity. As the imbalance between rate of heat production and heat dissipation determines the magnitude of heat storage and increases in core temperature (Kenny and Jay, 2013) athletes with tetraplegia demonstrate continual increases in body temperature during continuous submaximal exercise in both cool and warm conditions (Price and Campbell, 1999, 2006). For athletes with paraplegia, the reduced sweating capacity appears reasonably matched by the reduction in metabolic heat production as evidenced by similar increases in body temperature for athletes with high level lesion paraplegia (T1–T6) and lower level paraplegia (T7 and below) (Price and Campbell, 2003). Athletes with tetraplegia are thus considered to be at a greater risk of heat injury when compared to athletes with paraplegia who, in turn, have a greater risk of heat injury when compared to able-bodied athletes.

## Classical approaches to reduce heat strain

In preparation for competition in hot conditions, athletes generally consider heat acclimation (HA) or various cooling techniques to reduce heat strain and the subsequent risk of heat injury. Although HA is the key method recommended to optimize performance in hot conditions (Racinais et al., 2015) recommendations for reducing exertional heat injury for athletes with SCI are brief (Binkley et al., 2002) with no update in recent years (National Athletic Trainers' Association (NATA), 2014). Conversely, Griggs et al. (2015) recently reviewed cooling strategies in athletes with SCI concluding that due to the athletes reduced heat dissipation potential, using water sprays and cooling garments may be of great benefit. Furthermore, the majority of studies had not simulated true competitive situations and factors to optimize cooling potential within the constraints of competitive regulations have yet to be established. This is also true for cold slurry ingestion which may provide a useful heat sink for this population. Therefore, this article will focus on heat acclimation.

Heat acclimation refers to procedures to elicit favorable physiological adaptations to heat stress using artificial conditions whereas heat acclimatization involves natural conditions. Heat acclimation usually occurs as part of the athlete's preparation prior to traveling to holding camps where the more natural heat acclimatization can be undertaken prior to the competitive event. Heat acclimation (and heat acclimatization) classically occurs from repeated exposure to exercise in the heat over 5–14 days (Armstrong and Maresh, 1991). More recently, intermittent and shorter duration heat acclimation procedures as well as a “thermal clamp” method, where core temperature is increased during heat stress trials and maintained at a desired level, have been considered (Garrett et al., 2009; Chen et al., 2013). Nevertheless, whichever methods are utilized, HA and acclimatization result in a number of key physiological adaptations to improve heat dissipation (Armstrong and Maresh, 1991). In able-bodied athletes with an unaffected thermoregulatory system HA adaptations typically include; reduced deep body (“core”) temperature at rest as well as reduced deep body temperature, reduced skin temperature (and therefore reduced heat storage), increased skin blood flow and increased sweat rates at a given exercise intensity (Armstrong and Maresh, 1991; Armstrong, 2000; Lorenzo and Minson, 2010).

Sweating capacity and dynamic skin blood flow changes are well-known to be reduced or absent below the level of lesion in persons with SCI (Hopman, 1994; Price, 2006). Furthermore, similar deep body temperature and whole body sweat losses have been observed for both able-bodied upper body trained athletes and athletes with SCI during 60–90 min of arm crank exercise at the same relative exercise intensity (~60% peak oxygen uptake) in cool conditions (Price and Campbell, 1997, 1999). As these athletes with SCI would have had ~50% of their body surface area available for sweating these data suggest a greater sweat output per gland under the same exercise conditions and thermal strain. Subsequently, maximal sweat rates are potentially being achieved during exercise in cool environmental conditions by athletes with SCI (Price and Campbell, 2006). Alternatively, persons with SCI could demonstrate increased sweat gland activity and local sweat rates with heat acclimation. However, those persons with higher lesions levels (and lower whole body sweat rates) may not be able elicit a great enough increase in whole body sweat rate to affect core temperature responses. An athlete's remaining sweating capacity may subsequently be of key importance to heat acclimation success in this population.

## Heat acclimation studies in persons with SCI

Two studies have reported the responses of persons with SCI to a period of HA. Although it is difficult to conclude any specific HA outcomes in this population from such a small body of literature it is important to consider what is currently known to stimulate future research. Castle et al. (2013) examined a 7 day period of HA (33°C, 65% rh) in a mixed group of Paralympic shooters (*n* = 5) comprising of one athlete with tetraplegia (C4/5) two athletes with paraplegia (T9/10), one athlete with spina bifida (T6), and one athlete with Polio. Each day athletes undertook 60 min of heat exposure including 20 min of arm crank ergometry at 50 W followed by passive heat exposure or simulated shooting practice. Heat acclimation was partially evidenced as a reduction in resting and exercising aural temperature on day 7 compared to day 1 as well as decreased perceptions of effort, thermal strain, and increased plasma volume, thus supporting expected able-bodied adaptations.

Conversely, observations from our laboratory (Price et al., 2011) observed no typical HA responses for participants with tetraplegia (*n* = 5; C5–C7) or paraplegia (*n* = 5; T7-L1) also undertaking 7 days of HA, The protocol was similar to Castle et al. (2013) consisting of daily exercise in the heat (35°C, 40% rh) for 30 min at 50% peak aerobic power output followed by 30 min of passive recovery in the heat. Although the expected differences between groups for aural temperature were observed during exercise in the heat (Price and Campbell, 2003) no changes in aural temperature were observed between day 1 and day 7 for either group. A lack of HA may have been expected for the persons with tetraplegia with an absence of sweating, but not for the persons with paraplegia who demonstrated visible sweating capacity. It is possible that the exercise intensity, and thus body temperature stimulus, was not great enough to elicit heat acclimation however, the intensity was comparable to that of Castle et al. In addition both studies reported aural temperature as the deep body temperature estimate, with similar magnitudes of increase, so differences in deep body temperature site cannot solely explain the difference in results. Inter- and intra-individual variation in thermal responses, which are known to be large in the SCI population, along with differing lesion levels and disabilities may be key contributing factors between these studies observations.

Interestingly, reductions in perceived thermal strain on day 7 compared to day 1 with no change in aural temperature were observed for the group with paraplegia (Price et al., 2011). Such a response though may not be an advantageous adaptation as those individuals who cannot accurately assess their thermal status may be at a greater risk of heat injury (Goosey-Tolfrey et al., 2008). The group with tetraplegia showed no change in perceptions of thermal strain between day 1 and day 7, although thermal strain values were perceived at a similar level to the group with paraplegia even though aural temperatures during exercise were consistently greater. These responses suggest differences in the perception of thermal stimuli to those individuals with paraplegia and may potentially be due to much reduced surface area for afferent thermal information with tetraplegia when compared to paraplegia. Such responses should be examined in further detail. Although our data were collected from predominantly recreationally active, non-athlete participants such a population may represent athletes in sports where aerobic capacity and associated partial acclimation (as observed in able-bodied athletes; Piwonka et al., 1965) are not fully developed, coaches, or spectators. All of whom may be exposed to environmental stressors.

In addition to outdoor performances, indoor performances such as wheelchair basketball, rugby, and fencing may be of concern for some athletes. For example, a number of athletes, particularly those with tetraplegia, are unaware of the magnitude of rising body temperature during competitions undertaken in air-conditioned venues. Elevated “on court” body temperatures for some such players have been reported anecdotally by support staff and are similar to those observed during exercise in the heat or indeed the safety limits utilized in laboratory based thermoregulation studies. As heat acclimation has been shown to improve performance in cool conditions in able-bodied athletes (Lorenzo et al., 2010) this procedure may be of value for those athletes with lower level SCI and a significant sweating area, competing in indoor venues. However, the potential of reduced perceived thermal strain with no change in deep body temperature should always be considered. It should also be noted that other authors have observed no effect of heat acclimation on performance in cool conditions (Karlsen et al., 2015).

## Practical considerations

As can be gleaned from the above review, our knowledge of heat acclimation in athletes with SCI is considerably lacking. In addition, the wide range of individual responses to exercise in the heat, including skin temperature and sweating (Goosey-Tolfrey et al., 2008), makes general heat acclimation recommendations for this population difficult. It is possible that if athletes are educated regarding awareness of their thermal state during repeated exposure to heat and, importantly, have access to temperature monitoring devices for enhanced thermal safety assessment, such procedures may have performance benefits. As yet, few studies have considered performance aspects of thermal physiology in athletes with SCI so we are unfortunately unable to determine the efficacy of heat acclimation in this population with confidence. The same can also be stated for other groups of Paralympic athletes. As with most aspects of performance optimization, a considered individual approach needs to be taken with respect to environmental challenges. Appropriate medical back up and monitoring should always to available in such instances.

### Conflict of interest statement

The author declares that the research was conducted in the absence of any commercial or financial relationships that could be construed as a potential conflict of interest.

## Footnotes

^1^Worldweatheronline (2015). Available online at: http://www.worldweatheronline.com/Rio-De-Janeiro-weather-averages/Rio-De-Janeiro/BR.aspx (Accessed August 16, 2015).
